# Abnormal active myocardial relaxation by dipyridamole stress test in suspected early-stage heart failure with preserved ejection fraction: a retrospective exploratory analysis

**DOI:** 10.3389/fcvm.2026.1744083

**Published:** 2026-05-28

**Authors:** Gabriella Locorotondo, Monica Filice, Francesca Augusta Gabrielli, Giacomo Moretti, Laura Manfredonia, Antonella Lombardo, Gaetano Antonio Lanza, Leonarda Galiuto

**Affiliations:** 1 Department of Cardiovascular Sciences, Fondazione Policlinico Universitario A. Gemelli IRCCS, Rome, Italy; 2 Department of Diagnostic and Laboratory Medicine, Unity of Chemistry, Biochemistry and Clinical Molecular Biology, Fondazione Policlinico Universitario A. Gemelli IRCCS, Rome, Italy; 3 Catholic University of the Sacred Heart, Rome, Italy; 4 Department of Clinical and Molecular Medicine, Sapienza University of Rome, Sant’Andrea University Hospital, Rome, Italy

**Keywords:** active myocardial relaxation, cardiac time intervals, dipyridamole stress echocardiography, heart failure with preserved ejection fraction, myocardial performance

## Abstract

**Background:**

Early-stage heart failure with preserved ejection fraction (HFpEF) is like hypertensive heart disease (HHD) at resting echocardiography, but increases left ventricular (LV) filling pressure during stress. Coronary microvascular dysfunction (CMD) is a pathogenetic mechanism of HFpEF. Whether CMD actively increases LV filling pressure when LV stiffness is not yet established at rest remains unknown. LV mechanics were evaluated using dipyridamole stress echocardiography (DipSE) in suspected early-stage HFpEF.

**Methods:**

A total of 30 patients (mean age: 76 years; 80% female) with suspected early-stage HFpEF and 33 with HHD and normal NT-proBNP underwent DipSE. Systolic and diastolic indexes of LV myocardial function, particularly E/e′, isovolumic contraction and relaxation times (IVCT and IVRT), and ejection time (ET), were measured at rest and during stress. The myocardial performance index (MPI) was calculated as IVCT + IVRT/ET.

**Results:**

E/e′ ratio increased in suspected early-stage HFpEF patients (*p* < 0.001), but not in HHD patients. During stress, the IVRT reduced in HHD patients (*p* = 0.002), whereas remained significantly higher in suspected early-stage HFpEF patients (*p* < 0.001). Thus, MPI improved during DipSE in HHD patients (*p* = 0.005), but not in suspected early-stage HFpEF patients. Particularly, MPI was worse in suspected early-stage HFpEF patients with ST-segment depression than in those without it (*p* = 0.05).

**Conclusions:**

In suspected early-stage HFpEF, increased LV filling pressure during DipSE is associated with abnormal active myocardial relaxation. Myocardial performance is particularly impaired in patients with ST-segment depression during DipSE.

## 1 Introduction

Heart failure (HF) with preserved ejection fraction (HFpEF) represents up to 50% of HF cases at present, and its incidence is growing. The pathophysiology of HFpEF is complex and identifying different pathogenetic mechanisms could allow therapeutic strategies to be tailored on an individual basis. Abnormalities in left ventricular (LV) diastolic function (relying on impairment of active relaxation and/or increased stiffness), ventricular–arterial coupling, arterial stiffness, endothelial dysfunction, and chronotropic incompetence have been described. Each of these mechanisms may variably contribute to the pathogenesis of HFpEF in different patients, making this complex interplay difficult to diagnose and manage. In early-stage HFpEF, patients may experience exertional dyspnea, while diastolic function at rest may be normal or mildly altered. At this stage, HFpEF is not distinguishable from hypertensive heart disease (HHD) at resting echocardiography, but a significant rise in LV filling pressure and pulmonary artery systolic pressure (PASP) during exercise can be found. According to a proposed pathophysiological cascade, HFpEF results from a sequence of events initiated by a comorbidity-driven proinflammatory state associated with coronary microvascular dysfunction (CMD) (1). This, in turn, promotes LV hypertrophy, remodeling, fibrosis, and stiffness. Myocardial flow reserve, assessed via positron emission tomography at rest and during dipyridamole stress, is reduced in patients with HFpEF (2). CMD is associated with exacerbated diastolic dysfunction at rest (3). However, patients with significantly increased LV filling pressure at rest are affected by an advanced stage of HFpEF (4, 5), and the early stage of the disease remains poorly understood. In other terms, the “chicken and egg” question remains open, as it is challenging to establish the sequence of causality in the pathogenetic cascade; i.e., whether primary CMD leads to LV fibrosis, stiffening, and diastolic dysfunction or a post-inflammatory increase in myocardial fibrosis leads to secondary CMD. Given the capability of dipyridamole stress echocardiography (DipSE) to elicit abnormalities indicating CMD (6), we designed a single-center retrospective observational study to verify the features and pathophysiology of diastolic dysfunction at rest and during DipSE in a cohort of patients with potential early-stage HFpEF.

## 2 Materials and methods

An initial screening was carried out on an extensive database of patients referred to our Outpatient Cardiology Clinics from January 2020 to March 2024, to evaluate fatigue and/or breathlessness upon exertion via stress echocardiography. Among all patients undergoing stress echocardiography, those evaluated using DipSE were considered. The inclusion criteria were 1) age >18 years; 2) comprehensive echocardiographic assessment of diastolic and systolic LV function (see below for details); 3) LV ejection fraction (LVEF) ≥ 50%; and 4) available serum levels of NT-proBNP.

Patients were excluded if they had 1) a history of untreated significant epicardial coronary artery disease (CAD); 2) more than moderate valvular heart disease at rest or during DipSE; 3) wall motion abnormalities and/or LVEF < 50% at rest or during DipSE; 4) echocardiographic features of hypertrophic/dilated/infiltrative cardiomyopathy, arrhythmogenic cardiomyopathy, or pericardial constriction; and 5) suboptimal image quality. Moreover, patients experiencing angina or showing horizontal or downsloping ST-segment depression of 1 mm or more, measured 60-80 milliseconds after the J-point, were excluded if coronary angiography or computed tomography confirmed significant epicardial CAD.

The enrolled patients were further stratified based on the level of NT-proBNP (< or ≥125 pg/mL). Those with NT-proBNP ≥ 125 pg/mL were considered as affected by HFpEF if they fulfilled the criteria recommended by current guidelines, according to the simplified diagnostic approach (7): 1) symptoms and signs (pulmonary crackles and or lower-limb swelling) of HF; 2) LVEF ≥ 50%; 3) LV hypertrophy and/or left atrial (LA) enlargement, E/e′ at rest of at least 9 and pulmonary artery systolic pressure (PASP) ≥ 35 mmHg; and 4) NT-proBNP levels ≥125 pg/mL.

As suspected HFpEF in the early stage shows an echocardiographic phenotype very similar to that of HHD patients, we elected to compare patients with suspected early-stage HFpEF with patients showing a HHD phenotype, in order to detect any subtle differences between groups. Thus, patients with NT-proBNP < 125 pg/mL with a history of hypertension and evidence of LV hypertrophy and/or LA enlargement, featuring HHD were considered as a comparison group. The proposed HFA-PEFF scoring system (8) was applied to the 2 groups to score the probability of HFpEF better, taking into account different cut-offs for LA enlargement and NT-proBNP levels in patients with sinus rhythm and atrial fibrillation.

Each enrolled patient gave written informed consent to undergo DipSE. This study was conducted according to the Declaration of Helsinki on Human Rights.

NT-proBNP assessment was performed using a one-step sandwich chemiluminescent immunoassay.

Rest and stress echocardiography was performed using a commercially available Toshiba Artida (Tokyo, Japan) ultrasound machine, equipped with a 3.5 MHz probe. The stress protocol was carried out via intravenous infusion of 0.84 mg/Kg of dipyridamole over 10 min (0.56 mg/Kg over 4 min, followed by 4 min of no dose, and an additional 0.28 mg/Kg over 2 min), as previously validated (6). Both suspected early-stage HFpEF and HHD patients had to stop beta-blockers for 48 h and long-acting nitrates and calcium-channel blockers for 24 h before the test, and were recommended to avoid phylline-containing drugs or beverages for 24 h.

The echocardiographic images acquired both at rest and at stress peak were analyzed offline by an expert investigator (GL), who was blinded to the clinical data. Both biplane LA volume and LV mass (9) were indexed to body surface area (BSA) and expressed as LAVi and LVMi, respectively; they were considered increased if >34 mL/m^2^ and ≥115 g/m^2^ for males and ≥95 g/m^2^ for females, respectively (7). The LV diastolic function assessment was based on the E/A ratio and mean E/e′ratio values. Tricuspid annular plain systolic excursion (TAPSE) was measured as an index of right-ventricular systolic function at baseline. In contrast, the peak systolic longitudinal velocities for the interventricular septum (septal wall S′) on pulsed-wave tissue Doppler imaging (PW-TDI) and global longitudinal strain (GLS) on two-dimensional speckle-tracking analysis were considered as indices of LV longitudinal contractile function. As previously described in (10), the isovolumic contraction time, isovolumic relaxation time, and ejection time (IVCT, IVRT, and ET, respectively) were measured on PW-TDI and used to calculate the LV myocardial performance index (MPI) (11). MPI values were considered normal when lower than 0.39 (11), with greater values indicating progressively severe impairment of LV performance. Finally, the recorded tricuspid regurgitant (TR) peak velocities and inferior cava vein dimensions and collapsibility were used to derive the PASP value.

Clinical outcome was assessed by telephone calls. End-points included all-cause mortality and hospitalization for HF. In case of death, the cause was established from relatives and, when available, consulting electronic medical records.

### 2.1 Statistical analysis

All analyses were performed using the SPSS Statistics software (version 20.0) for Windows. Continuous variables are expressed as means ± standard deviations (SDs) if normally distributed, or medians and interquartile ranges (IQRs) if non-normally distributed, as assessed by Kolmogorov–Smirnov test. Categorical variables are presented as percentages. Intra-observer variability was tested for the main echocardiographic data. Comparisons between groups of continuous variables were done by Student's *t*-test or Man-Whitney test, as indicated. The chi-square test was applied to compare categorical variables. Pearson's or Spearman's correlation coefficients were used to assess the correlation between data for normally and non-normally distributed variables, respectively. Intra-group and inter-group comparisons between rest and stress data were performed using two-way ANOVA for repeated measures, and results were adjusted for potentially confounding covariates. A forward stepwise multivariable logistic regression analysis was carried out to identify independent predictors of suspected early-stage HFpEF diagnosis. To this aim, only clinical and echocardiographic variables showing a significant association with suspected early-stage HFpEF at standard statistical analyses were progressively included in the model according to their odds ratio, but maintained only when *p* < 0.05. Moreover, in patients with suspected early-stage HFpEF, univariate and multivariate Cox regression analysis was carried out to identify independent echocardiographic predictors of clinical outcomes: particularly, echocardiographic data during stress and their variation between rest and stress (calculated as Delta and expressed as percentage) were taken into account as potential predictors of outcome. Statistical significance was defined as *p* < 0,05 (2-sided). A *post-hoc* statistical power calculation was performed to check for reliability of results.

## 3 Results

### 3.1 General characteristics of study population

**Figure 1:**
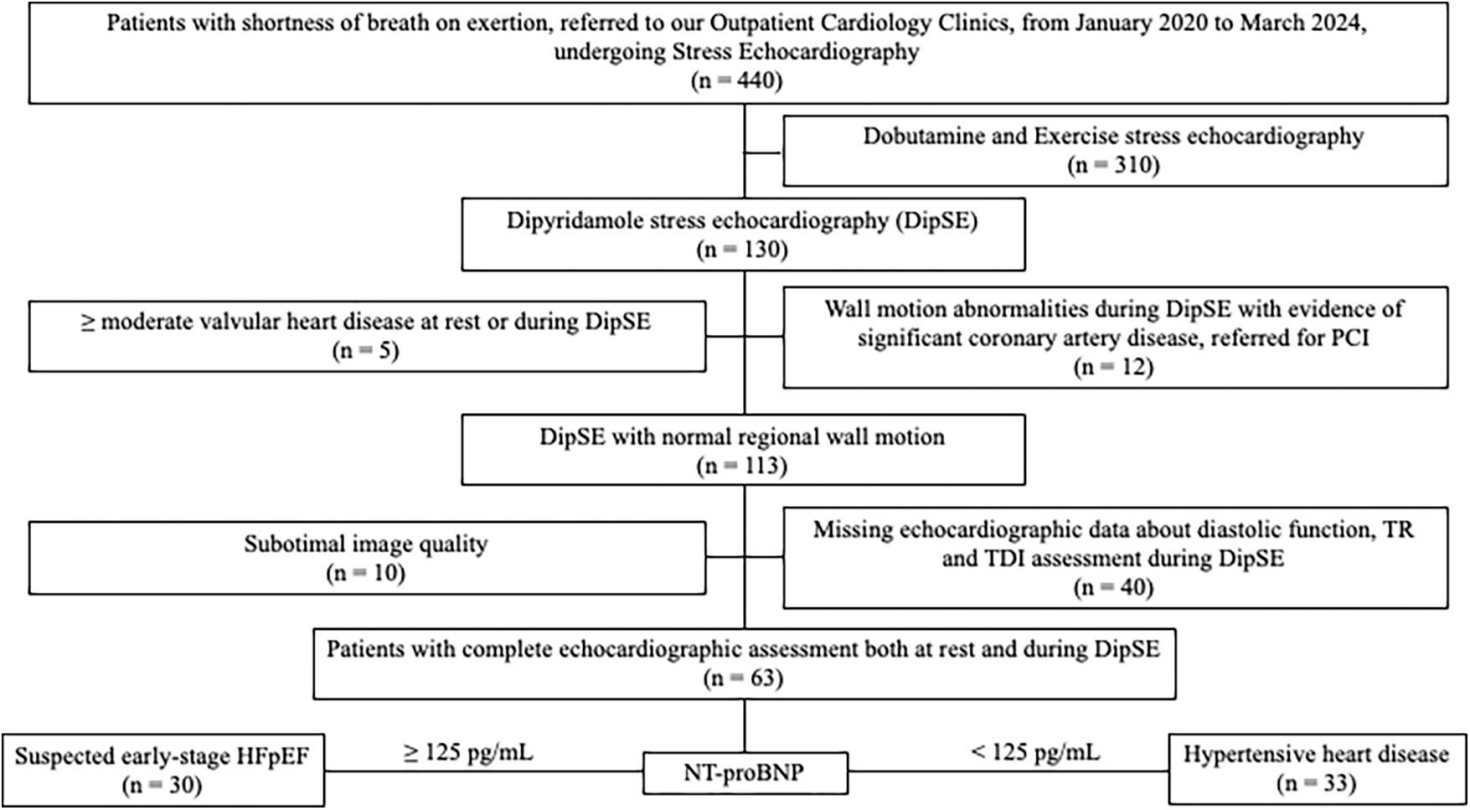
Study flowchart. DipSE, dipyridamole stress echocardiography; HFpEF, heart failure with preserved ejection fraction; PCI, percutaneous coronary intervention; TDI, tissue Doppler imaging; TR, tricuspid regurgitation.

Out of 200 patients initially selected, only 63 subjects constituted our final study population (Figure 1). The clinical characteristics are shown in Table 1. The intra-observer variability for all echocardiographic measures was lower than 5%. Subjects with NT-proBNP < 125 pg/mL showed a similar LV mass but smaller LA than patients with suspected early-stage HFpEF, thus presenting a HHD state. According to the allocation of patients in the two study groups, NT-proBNP ranged between a minimum of 51 pg/mL and a maximum of 110 pg/mL in the HHD group, and between a minimum of 129 pg/mL and a maximum of 1,360 pg/mL in the suspected early-stage HFpEF group (*p* < 0.001). Although the resting E/e′ values significantly differed between the two study groups, they showed some overlap, thus confirming that suspected HFpEF was in an early stage when the LV filling pressure at rest was normal or mildly altered. Notably, HFA-PEFF scores significantly differed between suspected early-stage HFpEF and HHD patients. As expected, patients with suspected early-stage HFpEF were older, showed higher prevalence of atrial fibrillation, dyslipidaemia, smoking habits, pulmonary disease, and exhibited greater uptake of calcium-channel blockers than patients with HHD.

**Table 1:** General characteristics of the study population.

	Total Population	Suspectd early-stage HFpEF	Hypertensive Heart Disease	p
	(*n* = 63)	(*n* = 30)	(*n* = 33)	
Demographics				
Age, yrs, mean ± SD	70 ± 10	76 ± 5	65 ± 11	**<0**.**001**
Female sex, *n* (%)	53 (84)	24 (80)	29 (88)	0.39
BMI, Kg/m^2^, mean ± SD	26 ± 4	27 ± 5	25 ± 3	0.08
BSA, m^2^, mean ± SD	1.7 ± 0.2	1.8 ± 0.1	1.7 ± 0.2	0.21
Cardiovascular risk factors				
Hypertension, *n* (%)	52 (82)	26 (87)	26 (79)	0.41
Diabetes mellitus, *n* (%)	5 (8)	4 (13)	1 (3)	0.13
Dyslipidemia, *n* (%)	37 (59)	22 (73)	15 (45)	**0**.**025**
Smoking, *n* (%)	12 (19)	10 (33)	2 (6)	**<0**.**001**
Previous hospitalization for CV causes				
Microvascular angina, *n* (%)	12 (19)	8 (27)	4 (12)	0.14
coronary syndrome, *n* (%)	2 (3)	0	2 (6)	0.17
Atrial fibrillation, *n* (%)	12 (19)	10 (33)	2 (6)	**0**.**006**
Medications				
Beta-blockers, *n* (%)	32 (51)	14 (47)	18 (54)	0.30
Calcium-channel blockers, *n* (%)	23 (36)	18 (60)	5 (15)	**0**.**001**
ACE inhibitors, *n* (%)	44 (70)	22 (73)	22 (67)	0.14
Diuretics, *n* (%)	15 (24)	10 (33)	5 (15)	0.14
Statins, *n* (%)	24 (38)	16 (53)	8 (40)	0.36
Nitrates, *n* (%)	0	0	0	/
Clinical status				
NYHA class				**0**.**005**
I, *n* (%)	21 (33)	4 (13)	17 (51)	
II, *n* (%)	30 (48)	18 (60)	12 (36)	
III, *n* (%)	12 (19)	8 (27)	4 (12)	
IV, *n* (%)	0	0	0	
HFA-PEFF points	2.8 ± 1.4	3.9 ± 1.0	1.8 ± 0.9	**<0**.**001**
Pulmonary disease, *n* (%)	14 (22)	10 (33)	4 (12)	**0**.**029**
Physical examination				
Systolic BP, mmHg, mean ± SD	131 ± 20	130 ± 21	132 ± 20	0.71
Diastolic BP, mmHg, mean ± SD	76 ± 11	77 ± 10	75 ± 12	0.46
Heart rate, bpm, mean ± SD	67 ± 11	65 ± 11	69 ± 10	0.14
Crackles, *n* (%)	8 (13)	6 (15)	2 (6)	0.097
Lower-limb swelling, *n* (%)	6 (10)	4 (13)	2 (6)	0.33
Laboratory values				
NT-proBNP, pg/mL, median (IQR)	107 (76–272)	281 (211–335)	76 (67–92)	**<0**.**001**
12-leads ECG at rest				
Pacemaker rhythm, *n* (%)	4 (6)	2 (7)	2 (6)	0.92
Atrial fibrillation, *n* (%)	2 (3)	2 (7)	0	0.13
Bundle branch block, *n* (%)	4 (6)	2 (7)	2 (6)	0.92
Echocardiography				
LVMi, gr/m^2^, mean ± SD	99 ± 25	95 ± 24	102 ± 25	0.65
LVEF, %, mean ± SD	65 ± 5	64 ± 4	66 ± 5	0.23
LAVi, mL/m^2^, mean ± SD	36 ± 8	39 ± 7	33 ± 8	**0**.**005**
TAPSE, mm, mean ± SD	22 ± 3	22 ± 4	21 ± 3	0.30

BMI, body mass index; BP, blood pressure; BSA, body surface area; ECG, electrocardiogram; IQR, interquartile range; HFA-PEFF, HFpEF, heart failure with preserved ejection fraction; LVEF, left ventricular ejection fraction; NYHA, New York Heart Association; SD,  standard deviation; TAPSE, tricuspid annular plane systolic excursion.

Bold values are statistically significant *p* values.

### 3.2 Echocardiographic analysis

**Figure 2:**
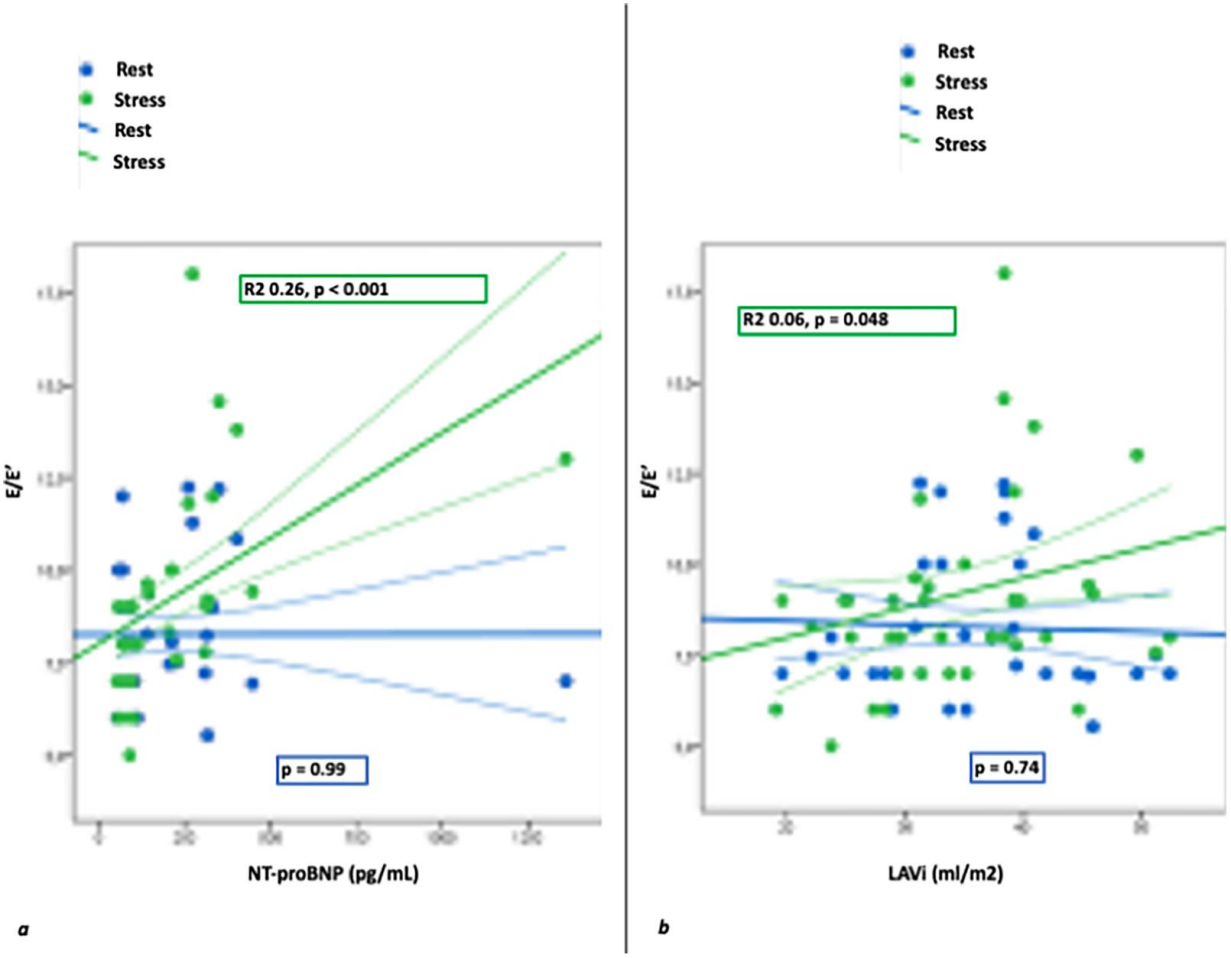
Correlation between E/e′ both at rest and during stress and NT-proBNP (panel a) and LAVi (panel a). Bold line indicates best fit; regular lines indicate 95% confidence interval. LAVi, left atrial volume indexed for body surface area.

Detailed echocardiographic measures of systolic and diastolic function at rest and during stress are reported in Table 2. During stress, 26 (87%) suspected early-stage HFpEF patients and 10 (30%) HHD patients (*p* < 0.001) experienced dyspnea. ST-segment depression was evident in 8 (27%) suspected early-stage HFpEF and 6 (18%) HHD patients (*p* = 0.39). With increasing heart rate, the cardiac cycle (i.e., RR interval) shortened from 921 ± 132 msec at rest to 742 ± 112 msec at stress peak (*p* < 0.001) in the former group and from 911 ± 141 msec at rest to 713 ± 118 msec at stress peak (*p* = 0.008) in the latter group (Table 2). In the overall study population, the stress E/e′ value, but not the rest E/e′ value, correlated with NT-proBNP (Figure 2a), similar to LAVi (Figure 2b).

**Table 2:** Echocardiographic characteristics in patients with HFpEF and hypertensive cardiomyopathy.

	Suspected early-stage HFpEF			HHD		
	(*n* = 30)			(*n* = 33)		
	Rest	Stress	p	Rest	Stress	*p*
RR interval, msec, mean ± SD	921 ± 132	742 ± 112	**<0** **.** **001**	911 ± 141	713 ± 118	**<0** **.** **001**
E/A, mean ± SD	0.82 ± 0.17	0.89 ± 0.21^a^	0.054	0.78 ± 0.25	0.76 ± 0.19	0.47
E/e′, mean ± SD	8.8 ± 2.0^a^	10.9 ± 2.9^b^	**<0** **.** **001**	7.8 ± 1.5	7.7 ± 1.1	0.59
PASP, mmHg, mean ± SD	28 ± 3	34 ± 5	**<0** **.** **001**	28 ± 4	33 ± 7	**<0** **.** **001**
Septal wall S′, cm/s, mean ± SD	7.6 ± 0.8	9.1 ± 1.3	**<0** **.** **001**	8.1 ± 1.2	9.6 ± 1.9	**<0** **.** **001**
IVCT, msec, mean ± SD	76 ± 20	68 ± 16	**0** **.** **010**	74 ± 18	66 ± 20	**<0** **.** **001**
IVRT, msec, mean ± SD	74 ± 23	71 ± 16^b^	0.44	69 ± 16	55 ± 16	**0** **.** **001**
relativeIVRT, %, mean ± SD	8 ± 2	10 ± 2^b^	**0** **.** **002**	8 ± 2	8 ± 2	0.87
ET, msec, mean ± SD	314 ± 36	303 ± 38	0.069	303 ± 31	287 ± 30	**0** **.** **003**
MPI, mean ± SD	0.47 ± 0.13	0.46 ± 0.10^a^	0.56	0.49 ± 0.13	0.43 ± 0.10	**0** **.** **005**
GLS, %, mean ± SD	−15 ± 5^b^	−20 ± 6^b^	**<0** **.** **001**	−16 ± 6	−19 ± 7	**<0** **.** **001**

ET, ejection time; HFpEF, heart failure with preserved ejection fraction; HHD, hypertensive heart disease; IVCT, isovolumic contraction time; IVRT, isovolumic relaxation time; MPI, myocardial performance index; PAPS, pulmonary artery systolic pressure; relativeIVRT, IVRT related to RR interval.

Al**l** comparisons between groups are adjusted for age, pulmonary disease, treatment by calcium-antagonist, dyslipidemia, smoking, previous atrial fibrillation, NYHA class, LAVi.

Bold values are statistically significant *p* values.

a *p* < 0,05 vs. HHD.

b *p* < 0,01 vs. HHD.

**Figure 3:**
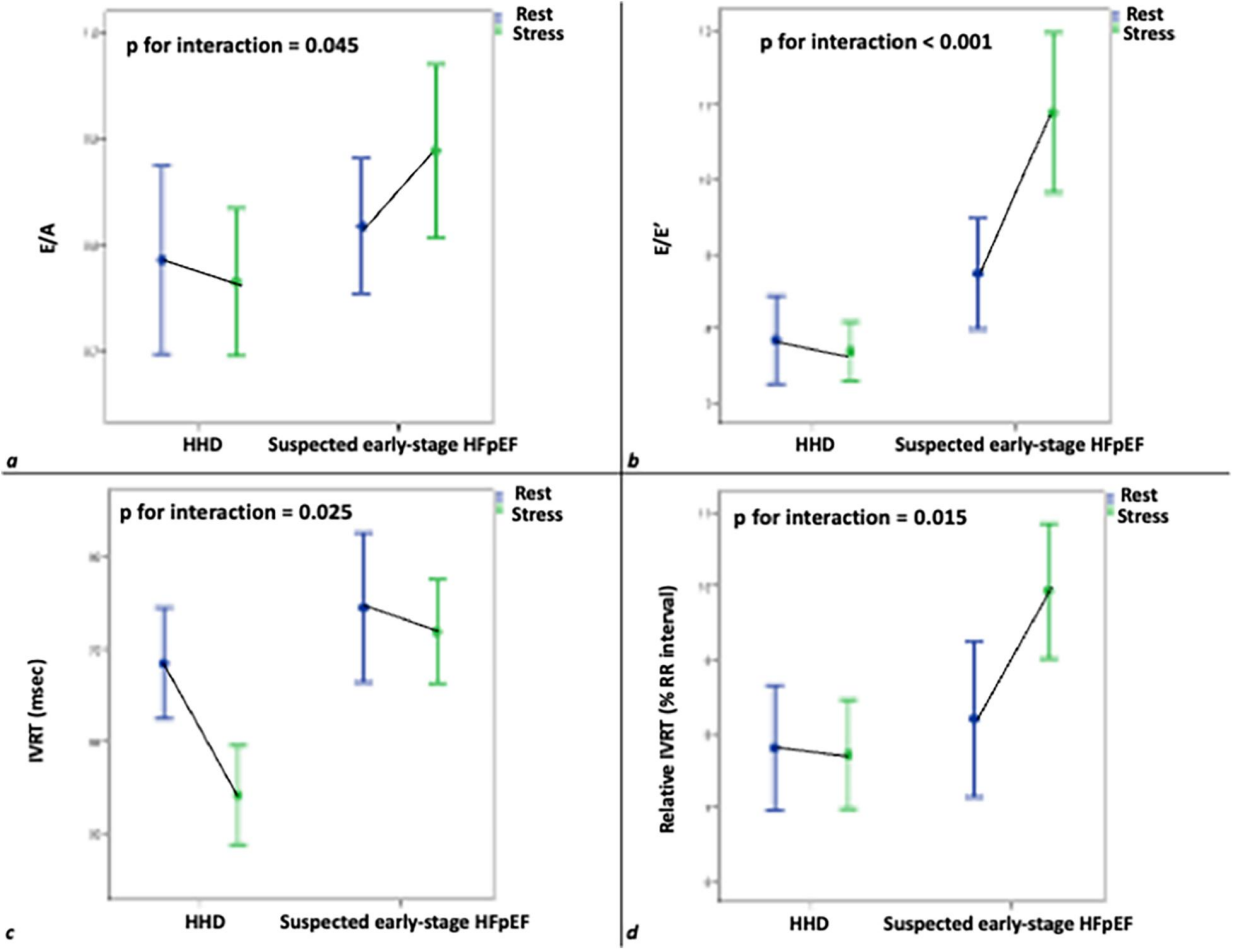
Comparison of rest and stress E/A (panel a), E/e′ (panel b), IVRT (panel c), and relative IVRT (panel d) values between suspected early-stage HFpEF and hypertensive heart disease patients. HFpEF, heart failure with preserved ejection fraction; IVRT, isovolumic relaxation time.

**Figure 4:**
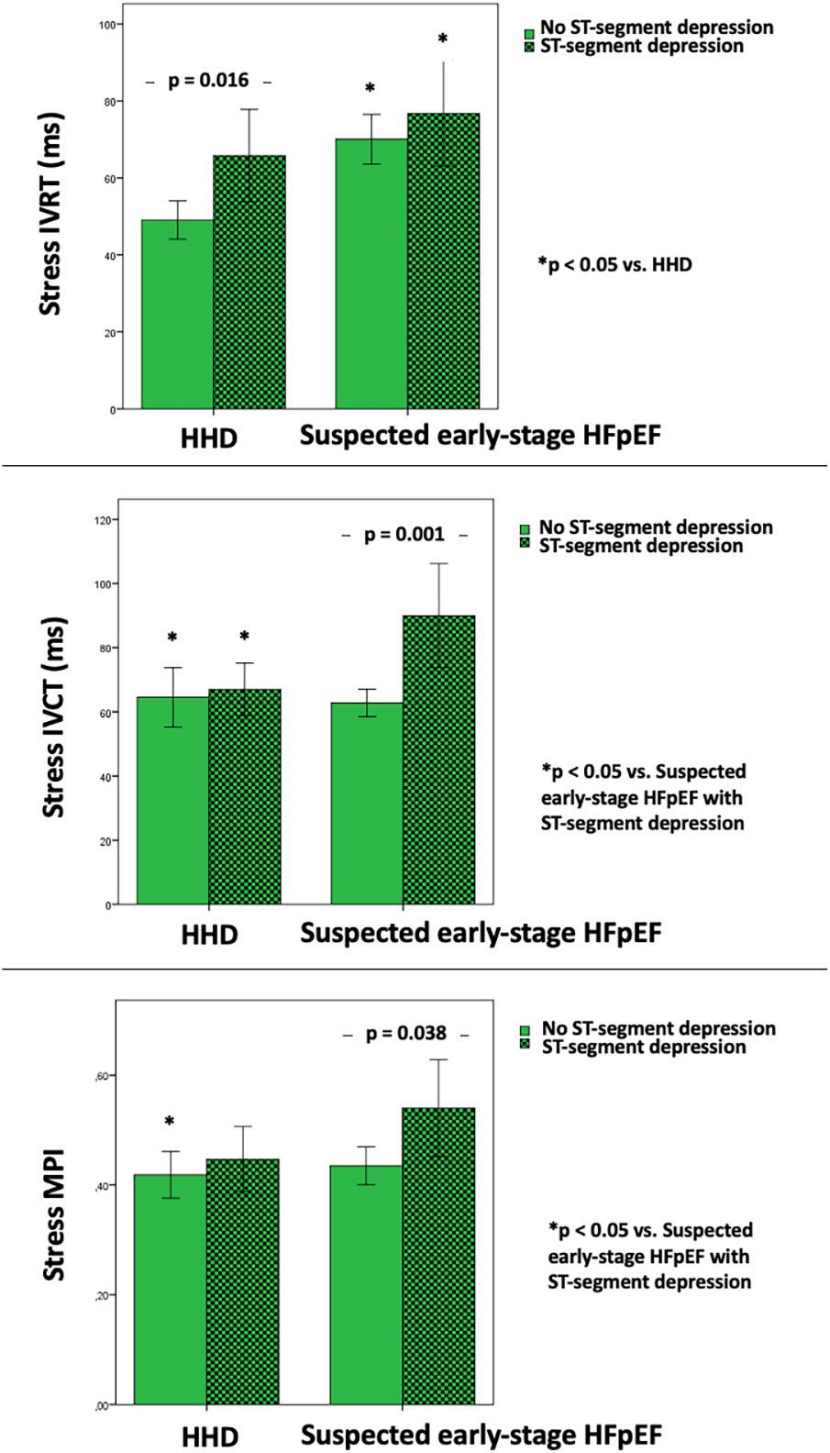
Comparison of stress IVRT (upper panel), stress IVCT (middle panel), and stress MPI (bottom panel) between patients with and without ST-segment depression in both groups. Subgroup sample sizes: suspected early-stage HFpEF patients with (8, 27%) and without (22, 73%) ST-segment depression; HHD patients with (6, 18%) and without (27, 81%) ST-segment depression. IVCT, isovolumic contraction time; IVRT, isovolumic relaxation time; MPI, myocardial performance index.

The E/A behaved differently in suspected early-stage HFpEF and HHD patients in response to dipyridamole (p for interaction: 0.045), although it did not significantly change during stress vs. rest in both groups (Figure 3a). The E/e′ significantly increased in the suspected early-stage HFpEF group but not in HHD patients (p for interaction: <0.001) (Figure 3b). The PASP increased in both groups (p for interaction: 0.23). Septal wall S′ velocities significantly increased during DipSE in both groups (p for interaction: 0.52). The GLS also improved in both groups, but its increase during stress was greater in HHD patients than in suspected early-stage HFpEF patients (p for interaction: 0.002). The IVCT similarly shortened in both groups (p for interaction: 0.079). The IVRT shortened during DipSE only in HHD patients (p for interaction: 0.025) and remained prolonged in suspected early-stage HFpEF patients (Figure 3c), so that it was significantly longer in suspected early-stage HFpEF patients than in HHD patients during stress (*p* < 0.001). This difference between groups was even more evident when the IVRT was expressed as a percentage of the RR interval, namely, the relative IVRT (Figure 3d). The MPI was altered in 60% of suspected early-stage HFpEF patients and 40% of HHD patients at rest. It remained unchanged in suspected early-stage HFpEF patients during DipSE (*p* = 0.56), whereas it significantly improved in HHD patients (*p* = 0.005 for stress vs. rest; p for interaction: 0.003). Finally, the stress IVRT tended to be generally higher in subjects developing ST-segment depression during DipSE than in those without ST-segment depression (Figure 4a). Furthermore, the stress IVCT and the stress MPI were worse in suspected early-stage HFpEF patients developing ST-segment depression than in those without ST-segment depression during DipSE (Figures 4b,c). At *post-hoc* calculation, statistical power to detect as significant (at *p* < 0.05) the differences between groups in the main variables of the study was >90%. At the multivariable analysis, stress MPI emerged as the only independent predictor of suspected early-stage HFpEF diagnosis (odd ratio 10.96, *p* = 0.031). The reason why stress IVRT and relative IVRT did not independently predict suspected early-stage HFpEF is probably because IVRT is already present in MPI formula.

### 3.3 Clinical follow-up

At a median follow-up of 46 months, all cause mortality occurred in 4 (13%) patients with suspected early-stage HFpEF and in 2 (6%) patients with HHD (*p* = 0.82), while hospitalization for HF occurred in 12 (40%) patients with suspected early-stage HFpEF and in 2 (6%) patients with HHD (*p* = 0.03). Results of univariate and multivariate Cox regression analysis for prediction of clinical events are reported in Table 3. Delta of PASP, E/e′, IVRT and relative IVRT all predicted adverse outcome at univariate regression analysis. At multivariate Cox regression analysis, however, no independent predictors were identified.

**Table 3:** Univariate and multivariate Cox regression analysis for prediction of major events (all-cause mortality or hospitalization for HF) in patients with suspected early-stage HFpEF.

	**Univariate**	**p**	**Multivariate**	**p**
	**Exp (B) (95% CI)**		**Exp (B) (95% CI)**	
Stress PASP (mmHg)	0.99 (0.88–1.11)	0.86		
Stress E/e′	0.83 (0.62–1.11)	0.21		
Stress IVCT (ms)	1.01 (0.98–1.05)	0.46		
Stress IVRT (ms)	1.00 (0.97–1.04)	0.80		
Relative stress IVRT	0.98 (0.79–1.21)	0.85		
Stress ET (ms)	1.00 (0.99–1.02)	0.52		
Stress MPI	3.08 (0.00–2,637)	0.74		
Stress GLS (%)	0.93 (0.83–1.04)	0.22		
Delta PASP (%)	**1.06** (**1.00–1.11)**	**0**.**035**	1.04 (0.98–1.11)	0.14
Delta E/e′ (%)	**0.96** (**0.93–0.99)**	**0**.**030**	0.99 (0.92–1.01)	0.42
Delta IVCT (%)	1.01 (0.98–1.04)	0.51		
Delta IVRT (%)	**1.03** (**1.00–1.06)**	**0**.**019**	1.01 (0.95–1.08)	0.68
Delta Rel IVRT (%)	**1.03** (**1.01–1.05)**	**0**.**007**	1.02 (0.97–1.07)	0.47
Delta ET (%)	0.98 (0.93–1.03)	0.46		
Delta MPI (%)	1.02 (1.00–1.05)	0.054		
Delta GLS (%)	1.05 (0.86–1.29)	0.61		

Bold values are statistically significant *p* values.

## 4 Discussion

In this study, for the first time, we assessed LV diastolic and systolic function at rest and during DipSE in patients with suspected early-stage HFpEF, compared with a group of patients with HHD, which at rest has an echocardiographic phenotype very similar to that of suspected HFpEF at the early-stage, by trying to identify differences between groups. The major results of our study, mainly involving female and elderly patients, are: 1) Suspected early-stage HFpEF patients showed an increased E/e′ ratio during DipSE; this was not found in patients with HHD. 2) Increased LV filling pressure was coupled with abnormalities in active myocardial relaxation during stress, while the LV longitudinal shortening reserve was preserved. 3) LV myocardial performance during DipSE was worse in patients with ST-segment depression than in those without. Although a direct assessment of coronary flow reserve was not performed to confirm microvascular ischemia, and a causal relationship cannot be stated, it seems conceivable that myocardial ischemia may at least in part contribute to the abnormal myocardial active relaxation and the abnormal myocardial performance detected during ST-segment depression. In this regard, dipyridamole stress-induced abnormalies in LV mechanics may appear before the establishment of increased resting LV filling pressure, which usually occurs due to myocardial fibrosis. Since conclusions of our study cannot be directly generalizable to both genders and across all ages, our findings should be rather considered as exploratory and hypothesis-generating and need to be confirmed in larger prospective studies.

### 4.1 Pathophysiology and clinical course of HFpEF

HFpEF is a complex cardiovascular disease with three different clinical stages, corresponding to different clinical disease severities (12). The first clinical stage is characterized by exertional dyspnea, without overt signs of volume overload. In such patients, clinical diagnosis relies on a combination of abnormalities in cardiac structure (LV hypertrophy and/or LA enlargement) and evidence of exercise-induced elevations in LV filling pressure. Early-stage HFpEF is not distinguishable from HHD at resting echocardiography: patients with LV hypertrophy secondary to arterial hypertension can develop HFpEF and have evidence of CMD (13). In the second clinical stage, patients present systemic volume overload and increased LV filling pressure at rest. The clinical course of HFpEF among different stages reflects worsening LV structure and mechanics over time, mainly characterized by a progressive increase in LV filling pressure and LV stiffness. From an ultrastructural point of view, such LV stiffness has been related to increased interstitial fibrosis. Moreover, an inverse relationship between myocardial fibrosis and microvascular density has been found in histological analysis of HFpEF patients. Autopsy findings of lower LV coronary microvascular density in HFpEF patients than controls have rapidly strengthened the hypothesis that CMD may promote the onset of the HFpEF syndrome through the development of fibrosis and hypertrophy. The existence of CMD has been confirmed *in vivo* in human studies evaluating myocardial blood flow and perfusion reserve using positron emission tomography (2). In the study of Srivaratharajah et al. (2), both the resting E/e′ ratio (14.7 ± 5.8) and the resting myocardial blood flow were significantly greater in patients with HFpEF than in normotensive controls. Meanwhile, Konerman et al. (3) found that patients with an E/e′ ratio of>15 at rest had the lowest value of myocardial flow reserve. Resting myocardial blood flow is high in patients with abnormal diastolic dysfunction, and diastolic function significantly declines when the coronary flow reserve is <2 (4); in such patients, elevated troponin can be detectable, thus suggesting that ischemia worsens global ventricular mechanics and dysfunction. All previous studies have enrolled patients with increased LV filling pressure at rest (i.e., the second stage of HFpEF). Some already established myocardial fibrosis may increase LV filling pressure at rest and might determine a low microvascular density with a compensatory increase in myocardial blood flow at rest. However, the “primum movens” in the pathophysiological cascade still needs to be determined. Our patient population belonged to the first type of clinical stage, where normal or mildly altered diastolic function at rest worsens during stress (14). The E/e′ ratio significantly increased compared with baseline, whereas the IVRT did not shorten substantially during stress, despite a reduction in the diastolic time with increasing heart rate. When the myocardial relaxation reserve is inadequate relative to the diastole shortening (15), the IVRT remains unchanged or even prolonged (16). As the IVRT relies on active myocardial relaxation and the impairment of LV performance during stress was mainly driven by abnormalities in the IVRT (16), this finding confirms the existence of active, rather than passive, changes in LV diastolic function. In the failing heart, maladaptive changes result in depressed intracellular Ca2+ cycling, such that contraction becomes slow and weak, and relaxation is prolonged; thus, early diastolic relaxation proceeds more slowly, explaining the prolongation of the IVRT. Different mechanisms may be responsible for such altered myocardial relaxation: myocardial steatosis, mitochondrial dysfunction due to an inflammatory state, and microvascular ischemia. Impairment of LV myocardial performance was more pronounced in our patients with ST-segment depression than in those without it during stress. Microvascular ischemia would be expected to cause a shift in energy metabolism from free fatty acids to glucose, with a consequent abnormal elevation in the myocardial triglyceride content (17).

### 4.2 Myocardial relaxation and stiffness in HFpEF

Impaired active relaxation seems predominant in HFpEF patients without increased myocardial fibrosis. In contrast, an increase in passive stiffness has been identified as the primary mechanism in patients with elevated extracellular matrix deposition (18). Although the E/e′ ratio is widely used as an indicator of mean LV filling pressure, it cannot discriminate between impaired relaxation and LV stiffness. Reduced systolic longitudinal shortening is a typical feature of HFpEF, and changes during stress may indicate LV stiffness (19). Recente evidences suggest that the ratio of transmitral early filling velocity to early diastolic strain rate (E/e′ SR) measured by speckle-tracking echocardiography, also considered a reliable marker of left ventricular filling pressure, is a powerful predictor of short-term outcome in patients with advanced HF with reduced ejection fraction, NYHA III-IV functional status, and pulmonary or systemic congestion requiring intravenous diuretics (20). In our study, early diastolic strain rate was not assessed, but we demonstrated the existence of longitudinal shortening reserve during stress. In patients with acute ischemic stroke, the increase of GLS may reflect compensatory myocardial hyperfunction, especially in the diastolic phase, due to sympathetic activation (21). In our patients, the existence of a longitudinal shortening reserve can exclude that structural fibrotic changes in myocardium had been already established. Finally, the abnormalities in active relaxation in our patients cannot be explained by an increased afterload, which usually occurs during exercise or hypertensive stress (22), because no significant increase in blood pressure during stress was registered. Notably, regarding the small number of males enrolled in both groups, no comparison between genders was made, so it is unclear whether our results are generalizable to males and females. However, HFpEF is known to occur predominantly in older women.

### 4.3 Role of dipyridamole as diastolic stressor in HFpEF

Most of the previous studies have used exercise stress to unmask abnormalities in diastolic function (23). Using DipSE, we found an increase in the E/e′ ratio and a further impairment of myocardial performance in patients showing ST-segment depression during stress. The rise in PASP was similar in suspected early-stage HFpEF and HHD patients. It may be related to the direct effect of dipyridamole on the pulmonary system, as endogenous adenosine acts as a powerful bronchoconstrictor. However, in patients without CAD, an abnormal thallium-201 lung uptake during dipyridamole is predictive of elevated filling pressures, probably reflecting an abnormal response to vasodilatation (24). Dipyridamole is known to increase myocardial contractility, probably via an adrenergic mechanism (25). In patients with a very low CAD risk, dipyridamole administration improved ventricular energetics, decreased end-diastolic and end-systolic volumes, and increased ejection fraction and ventricular–arterial coupling (26, 27). Simultaneously, it does not have any effect on stroke volume (27). Less robust data have been published about IVRT and diastolic function. In patients with a slow coronary flow at rest and a normal vasodilator response, orally administered dipyridamole normalized the IVRT and myocardial performance (28). Our suspected early stage HFpEF patients showed mildly altered diastolic function at rest, which further worsened during stress, mainly in patients with ST-segment depression, thus suggesting that slow coronary flow at rest and a reduced vasodilator response during stress may occur. Nevertheless, whichever is the exact pathophysiologic mechanism, the abnormal response of myocardial relaxation during DipSE was associated with a higher mortality and hospitalization for HF in our predominantly elderly female population with suspected early-stage HFpEF.

### 4.4 Limitations

Some limitations of our study should be acknowledged. First, our study included a rather small number of patients only, which mainly resulted from its retrospective nature.

Second the classification of patients as suspected early-stage HFpEF was based on subjective symptoms and biomarker measurements, rather than HFA-PEFF criteria (8), which might appear arbitrary. As stated by the guidelines, diagnostic performance of HF scores varies, as a substantial proportion of suspected HFpEF patients receive an intermediate likelihood. It has been further highlighted by recent papers (29, 30). The mean HFA-PEFF scores in our study were intermediate in suspected early-stage HFpEF patients and low in HHD patients.

Third, it might be objected that a HHD group, rather than a healthy subject control groups was used for comparison in our study. However, considering the retrospective nature of the study, it would have been impossible to identify a significant number of asymptomatic healthy individuals that underwent DipSE at our Center. Furthermore, our control groups was accurately selected among patients undergoing stress echocardiography due to the occurrence of dyspnea on effort. Moreover, the purpose of our study was to explicitly try to identify early pathophysiological mechanisms featuring HFpEF and able to distinguish it from a HHD.

Fourth, it may be argued that abnormalities in the relaxation process prolong the IVRT, whereas further worsening of LV diastolic function (i.e., pseudonormal) provokes shortening of the IVRT at rest due to an increase in LV filling pressure. However, previous data from cardiac catheterization at rest and during exercise in patients with newly diagnosed HFpEF have demonstrated that, although the diastolic filling time was reduced with increases in heart rate, the enhancement in relaxation with exercise was inadequate to compensate for the rate-related shortening of the diastolic filling period. Thus, the proportion of diastole elapsed before the estimated complete relaxation increased, suggesting an insufficient relaxation reserve relative to the shortening of diastole (15). A direct comparison of flow augmentation between DipSE and leg-raising or fluid challenge has never been performed, but increased preload might be the pathophysiological mechanism explaining our results. Unfortunately, antegrade flow throughout the mitral valve at rest and during stress was not routinely assessed because it is not recommended.

Fifth, this study lacked a direct measurement of coronary flow reserve (CFR) or myocardial flow reserve (MFR). However, previous studies largely showed the presence of a CMD-related impairment of coronary blood flow in response to dipyridamole in HFpEF patients, after excluding obstructive CAD, as in our patients (6). Notably, in the iPOWER sub-study carried out in women with angina, left ventricular ejection fraction >45%, and no obstructive CAD (31), Pena et al. gained results similar to ours: CMD was associated with a higher E/e′ ratio, a longer IVCT, and a higher MPI. Additionally, in a multivariable analysis, a longer IVCT and a higher MPI remained associated with CMD. Finally, the pathophysiology of HFpEF is too complex to be explained only with our results, which did not account for ventricular-arterial uncoupling, arterial stiffness, endothelial dysfunction, as well as the roles of obesity and pulmonary disease. For the retrospective nature of the study, we intended verifying whether a vasodilation stress test may elicit diastolic dysfunction induced by microvascular ischemia, in an early stage of the disease. How microvascular ischemia might potentially worsen other peripheral dysfunction cannot be elucidated by our results.

Finally, we compared a large number of echocardiographic, as well as clinical, parameters between the 2 groups. The risk that we only casually found as significant some difference (type I error) cannot be excluded, which suggests caution in the interpretation of the data and need of confirmation in further studies.

Most patients included in the study (84%) were females and elderly (mean age 76 years). Thus, whether our data can be generalized to the whole population of early HFpEF patients, including a sizeable proportion of males and younger patients, needs assessment in further studies. It should be noticed, however, that our sample reflects the predominant prevalence of female and elderly patients in the population of patients with HFpEF.

## 5 Conclusions

In patients suffering from effort dyspnea, screening for diastolic dysfunction is desirable but limited by reduced exercise capacity. When challenged with DipSE, predominantly female elderly patients with suspected early-stage HFpEF may experience an increase in the E/e′ and abnormal active myocardial relaxation without signs of LV stiffness at rest. Myocardial performance seems particularly impaired in patients with ST-segment depression during stress, thus suggesting that an abnormal myocardial relaxation, contributed at least in part by myocardial ischemia, might lead to stress-induced diastolic dysfunction. The limited sample size and retrospective nature of our study, however, suggest that further data are needed to confirm our findings. This is the first study using DipSE as a diastolic stress test in patients with features of suspected early-stage HFpEF: although DipSE hold promise as a screening tool for early HFpEF, it will require further clinical validation.

## Data Availability

The raw data supporting the conclusions of this article will be made available by the authors, without undue reservation.
